# A Systematic Study of RNAi Effects and dsRNA Stability in *Tribolium castaneum* and *Acyrthosiphon pisum*, Following Injection and Ingestion of Analogous dsRNAs

**DOI:** 10.3390/ijms19041079

**Published:** 2018-04-04

**Authors:** Min Cao, John A. Gatehouse, Elaine C. Fitches

**Affiliations:** Department of Biosciences, Durham University, Durham DH1 3LE, UK; j.a.gatehouse@durham.ac.uk (J.A.G.); E.C.Fitches@durham.ac.uk (E.C.F.)

**Keywords:** dsRNA stability, RNAi, exo-nucleases, endo-nucleases, flour beetle (*Tribolium castaneum*), pea aphid (*Acyrthosiphon pisum*)

## Abstract

RNA interference (RNAi) effects in insects are highly variable and may be largely dependent upon the stability of introduced double-stranded RNAs to digestion by nucleases. Here, we report a systematic comparison of RNAi effects in susceptible red flour beetle (*Tribolium castaneum*) and recalcitrant pea aphid (*Acyrthosiphon pisum*) following delivery of dsRNAs of identical length targeting expression of V-type ATPase subunit E (*VTE*) and inhibitor of apoptosis (*IAP*) genes. Injection and ingestion of *VTE* and *IAP* dsRNAs resulted in up to 100% mortality of *T. castaneum* larvae and sustained suppression (>80%) of transcript levels. In *A. pisum*, injection of *VTE* but not *IAP* dsRNA resulted in up to 65% mortality and transient suppression (ca. 40%) of *VTE* transcript levels. Feeding aphids on *VTE* dsRNA reduced growth and fecundity although no evidence for gene suppression was obtained. Rapid degradation of dsRNAs by aphid salivary, haemolymph and gut nucleases contrasted with stability in *T. castaneum* larvae where it appears that exo-nuclease activity is responsible for relatively slow digestion of dsRNAs. This is the first study to directly compare RNAi effects and dsRNA stability in receptive and refractory insect species and provides further evidence that dsRNA susceptibility to nucleases is a key factor in determining RNAi efficiency.

## 1. Introduction

The use of RNA interference (RNAi) to suppress the expression of target genes in insects is proven as a research technique to elucidate gene function [1,2]. In 2007, a breakthrough paper by Baum et al. [3] demonstrated potential for the exploitation of RNAi as an elegant, target specific, strategy for the control of corn rootworm (*Diabrotica virgifera virgifera*: Coleoptera) larvae using genetically modified (GM) plants. Successful induction of RNAi effects through injection or feeding dsRNAs has been achieved in many insects from different orders including species belonging to Coleoptera [3,4,5,6,7,8,9,10], Hemiptera [11,12,13,14], Lepidoptera [15,16,17,18], Diptera [19], Dictyopteran [20,21,22,23], Hymenoptera [24] and Isoptera [25]. Delivery via microinjection of dsRNAs into the haemocoel has generally been found to induce greater, more consistent gene knock-down and lethality as compared to feeding dsRNAs [26,27,28]. However, considerable variability in responses to ingested dsRNAs currently limits application of this technology as a general strategy for crop protection [26,27,29,30,31]. Whilst many of the core RNAi genes appear to be conserved amongst insects, a multitude of factors including developmental stage, tissue type, target gene, selected region within the target gene, as well as the length and amount of introduced dsRNA have been shown to influence RNAi effects [7,8,26,27,28,30,32]. Further complexity is provided by gaps in understanding of the relative stability of dsRNAs in vivo and the mechanisms underlying gene uptake into cells. 

Robust and systemic RNAi effects in the red flour beetle *Tribolium castaneum* are well documented [5,7,33,34,35] with early evidence for transgenerational RNAi demonstrated by Bucher et al. [4]. Strong RNAi effects have also been reported for other coleopteran species including western corn rootworm (*Diabrotica virgifera*) [3,8]; colorado potato beetle (*Leptinotarsa decemlineata*) [3,36,37,38]; African sweet potato weevil (*Cylas puncticollis*) [39]; Asian longhorn beetle (*Anoplophora glabripennis*) [40] and small hive beetle (*Aethina tumida*) [41]. The injection and ingestion of dsRNAs commonly induces significant levels of gene-knock down and systemic RNAi responses in coleopteran species [3,5,7,8,33,34,35,36,37,38,40].

RNAi effects in Hemipteran species are extremely diverse, ranging from no phenotype to significant mortality and from very low to complete gene knock-down [30,42]. Variability in RNAi effects have even been observed when considering the same target gene within a single species. For example, Whyard et al. [33] reported significant levels of mortality for pea aphids (*Acyrthosiphon pisum*) fed on artificial diet containing dsRNA targeting expression of V-type ATPase subunit E (LC50 0.00344 mg/g diet), and a 30% reduction in target mRNA levels. By contrast, Christaens et al. [43] reported no phenotype or gene down-regulation for pea aphids injected with or fed on diet containing comparable amounts of dsRNA targeting expression of V-type ATPase subunit E. Experiments where hemipteran species are fed on transgenic plants expressing dsRNA appear to have produced more consistent results, although in such studies insects are exposed to short interfering (si)RNAs that have been processed from dsRNA *in planta*. Pitino et al. [13] reported up to 60% down-regulation of *MpC002* (expressed in salivary glands) and *Rack-1* (expressed in gut) expression in pea aphids, and were able to show reduced fecundity after feeding aphids on ds-RNA transgenic plants. Similarly, Zha et al. [14] reported knock-down of two RNAi pathway genes in rice brown plant hopper (*Nilaparvata lugens*) fed on transgenic dsRNA rice. Abdellatef et al. [44] reported silencing of a salivary sheath protein and phenotypic effects in cereal (*Sitobion avenae*) aphids fed on transgenic barley expressing siRNAs with transgenerational effects observed for up to 7 generations.

To successfully induce RNAi, introduced dsRNAs must remain in a non-degraded state for a sufficient period to allow dsRNA to be taken up by insect cells. Garbutt et al. [45] were the first to show that dsRNA persisted for up to 24 h in haemolymph extracted from cockroach (*Blattella germanica*) known to be susceptible to RNAi, whereas rapid dsRNA degradation (1 h) was observed in the haemolymph of the refractory tobacco hornworm (*Manduca sexta*). Rapid degradation of environmental dsRNA by extracellular ribonucleases in the haemolymph and gut is increasingly recognised as a key factor in determining RNAi efficiency in a number of different insect species [31,45,46,47,48,49,50]. This is particularly key for hemipteran species where extra-oral salivary degradation of dsRNAs provides an additional barrier to cellular uptake [43,51,52,53]. 

Here we have conducted a direct comparison of the efficiency of RNAi in the coleopteran *T. casteneum* with the hemipteran *A. pisum.* Double stranded RNAs of identical length, targeting *V-ATPase subunit E* (*VTE*) and *Inhibitor of apoptosis* (*IAP*) genes, have been administered by injection and feeding. Exposure to comparable doses of dsRNAs (by insect weight) has enabled a direct comparison of RNAi induced effects on survival and gene expression in the different insects. Our results show systemic RNAi responses in *T. castaneum* larvae by injection and feeding, as compared to a relatively weak and transient gene dependent response in *A. pisum*. Comparative in vitro experiments provide further evidence to suggest that dsRNA degradation by extracellular ribonucleases plays a critical role in determining the poor efficiency of RNAi in *A. pisum*. By contrast, relatively slow degradation of dsRNA by exonucleases is suggested to be a major factor in facilitating consistent RNAi effects in *T. castaneum*. 

## 2. Results

### 2.1. Expression of VTE and IAP during the Development of A. pisum and T. castaneum

**Figure 1:**
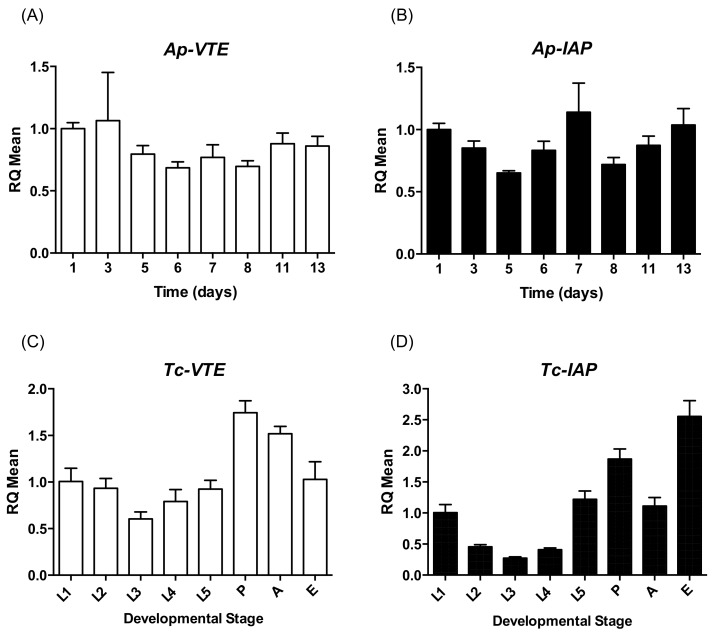
Expression of *V-ATPase subunit E* (*VTE*, shown in white column) and *inhibitor of apoptosis* (*IAP*, shown in black column) genes throughout the life cycle of (**A**,**B**) *A. pisum* (*Ap*) and (**C**,**D**) *T. castaneum* (*Tc*) by quantitative PCR. For *Ap*, day 1 corresponds to the nymphal stage with analysis following development at days specified until the onset of nymph production at day 13. Developmental stages for *Tc* are L1 = 1st–2nd instar, L2 = 2nd–3rd instar, L3 = 3rd–4th instar, L4 = 4th–5th instar, L5 = 5th–6th instar (pre-pupal stage), P = pupa, A = adult, E = egg. RQ set to 1.0 for Day 1 or L1 samples. Error bars depict ±SD of the mean for 3 technical replicates (*n* = 5 insects or 30 mg eggs per replicate).

Target transcripts were present at similar levels throughout the life cycle of *A. pisum* (Figure 1A) although *IAP* mRNA levels were found to be more variable (Figure 1B) as compared to *VTE*. For *T. castaneum*, the expression of *VTE* and *IAP* genes appears to be more dependent upon developmental stage. V-ATPase subunit E transcript levels were almost 2 times greater in pupae and adults, as compared to egg and larval stages (Figure 1C). Inhibitor of apoptosis mRNA levels are highest in beetle eggs, dropping to lower levels during larval development before rising again during the pre-pupal and pupal stages (Figure 1D).

### 2.2. Effect of Injected dsRNAs on Phenotype and Target Gene Expression

**Figure 2:**
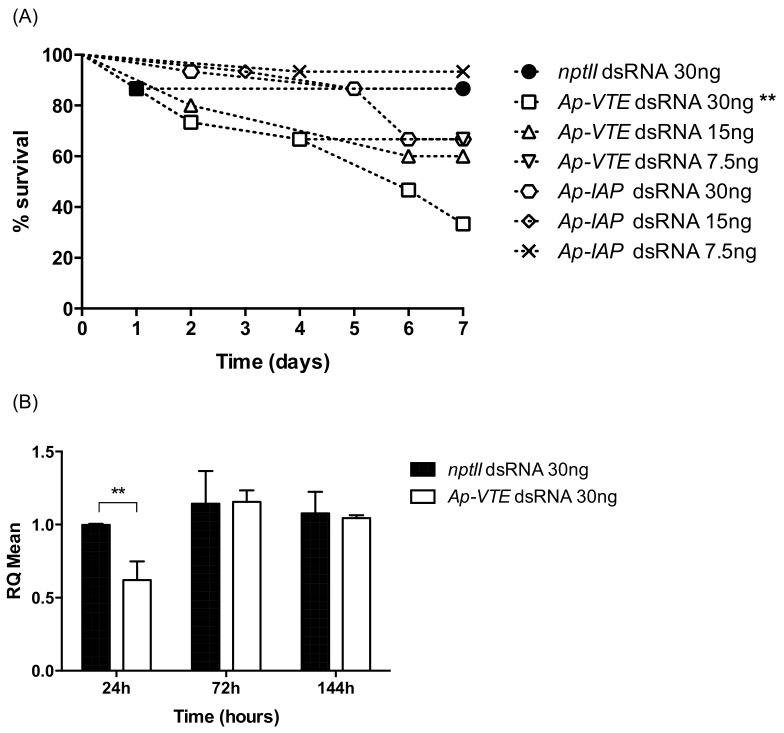
(**A**) Aphid survival after injection of *Ap-VTE* or *Ap-IAP* dsRNAs (*n* = 15 per treatment). (**B**) Relative expression of *Ap-VTE* mRNA in injected aphids. RQ set to 1.0 for *nptII* control 24 h treatment. Error bars indicate the ±SD of the mean from two independent biological replicates (*n* = 5 insects per treatment), each with three technical replicates. Asterisks depict significant differences ** *p <* 0.01 in survival or mean mRNA levels, as compared to controls.

Aphids injected with 30 ng control dsRNA showed a small decrease in survival (14%) over an assay period of 7 days (Figure 2A). Aphids injected with *VTE* dsRNA exhibited a dose dependent reduction in survival (33%, 60% and 77% reduction in survival for doses of 7.5, 15 and 30 ng, respectively), although effects were only significantly different to control injected insects at the highest dose of 30 ng dsRNA (Figure 2A; *p* < 0.01, Analysis of Variance (ANOVA) Log-rank Mantel-Cox). By contrast, survival of aphids injected with the highest 30 ng dose of *IAP* dsRNA was reduced by less than 15% as compared to the control *nptII* group. By the end of the assay (corresponding to day 12 of the life cycle), 3–5 nymphs per adult were produced from control treatment and *IAP* dsRNA injected treatments whereas no nymphs were produced by surviving aphids injected with *VTE* dsRNA. 

Quantitative PCR analysis of target gene mRNA levels was conducted to investigate if the observed mortality of injected aphids was attributable to gene suppression. Injections of 30 ng *VTE* dsRNA (equivalent to 37.5 ng dsRNA/mg aphid) significantly reduced target transcript levels (approx. 38% relative to the control treatment; *p* < 0.01; students *t*-test) 24 h post injection (Figure 2B). However, comparable levels of mRNA in control and *VTE* dsRNA injected aphids 72 and 144 h post-injection (Figure 2B) indicated that gene suppression effects were transient. Quantitative PCR analysis of *IAP* mRNA levels after injections of 30 ng of target dsRNA did not show any evidence of gene knock-down with transcript levels similar to control injected aphids 24 h, 72 h, and 144 h post injection.

**Figure 3:**
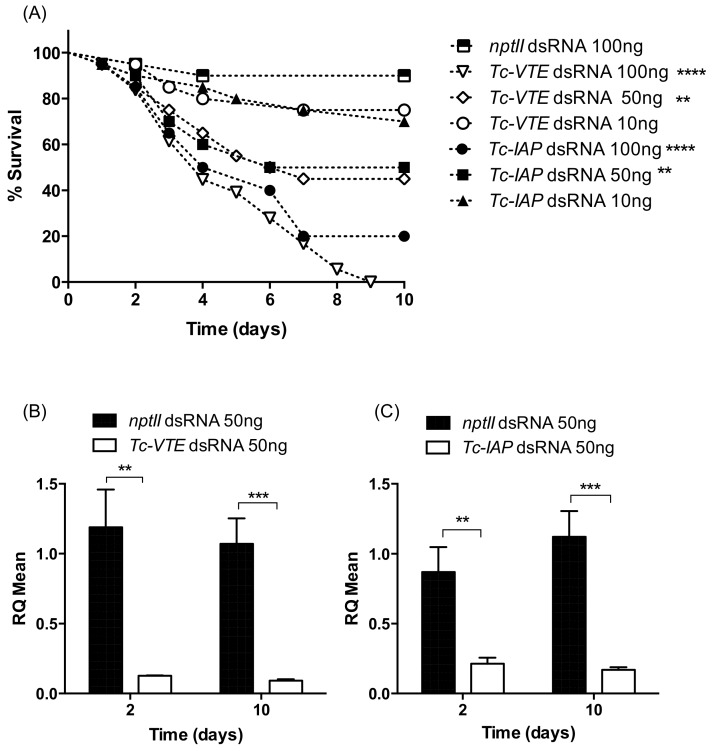
(**A**) *T. castaneum* survival after injection of *Tc-VTE* and *Tc-IAP* dsRNAs into pre-pupal stage larvae (*n =* 20 per treatment). Relative expression of (**B**) *Tc-VTE* and (**C**) *Tc-IAP* mRNAs after injection of target dsRNAs into pre-pupal stage larvae. Error bars indicate ±SD of the mean from three biological replicates (*n =* 5 insects per treatment), each with three technical replicates. RQ set to 1.0 for *nptII* control day 2 biological replicate 1. Asterisks depict significant differences **** *p <* 0.0001; *** *p <* 0.001 and ** *p <* 0.01 in survival or mean mRNA levels (as compared to controls).

Pre-pupal *T. castaneum* larvae injected with *VTE* or *IAP* dsRNA exhibited similar dose dependent reductions in survival over an assay period of 10 days (Figure 3A). At the highest dose of 100 ng (equivalent to 34.4 ng dsRNA/mg larvae) 100% and 80% mortality was recorded respectively, for *VTE* and *IAP* injected insects (*p <* 0.01; ANOVA Log-rank Mantel Cox tests). Effects on survival were also significant, as compared to the control treatment, at the lower 50 ng injection dose where approx. 50% mortality was recorded for both dsRNA treatments. 

The expression of *VTE* and *IAP* genes in *T. castaneum* after injection of dsRNAs was assessed by qPCR. In both cases larvae injected with 50 ng target dsRNA (equivalent to 17.2 ng dsRNA/mg insect weight) showed significant >85% reductions in mRNA levels, relative to control treatments (Figure 3B,C; *p* < 0.01; students *t*-tests). Analysis of samples taken 10 days after injection showed that mRNA levels were comparable to those recorded 2 days post injection confirming the persistence of gene suppression over time. Overall, the injection of *VTE* resulted in a 16-fold reduction in mRNA levels as compared to an 8-fold reduction for larvae injected with *IAP* dsRNA suggesting that the former was approx. 2× more effective at inducing gene knock-down. As the dsRNAs were designed to be of the same length, the *VTE* dsRNA was also more effective than *IAP* on a molar basis (i.e., effect per molecule of dsRNA). 

### 2.3. Oral Delivery of dsRNA 

#### 2.3.1. Stability of dsRNAs in Insect Diets 

**Figure 4:**
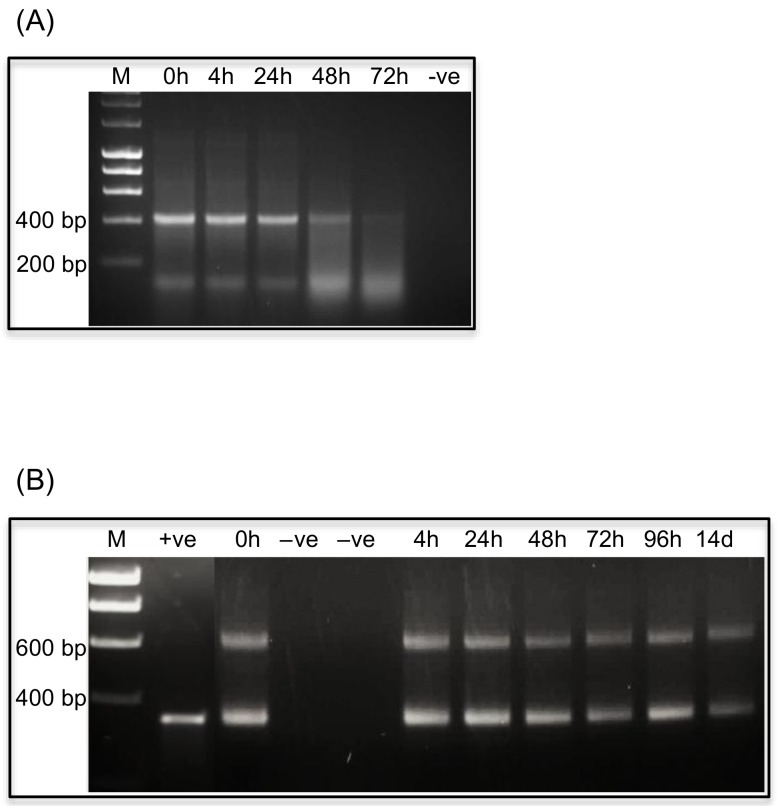
dsRNA stability over time in (**A**) aphid diet and (**B**) in wheat flour discs in the presence of feeding insects. For *Ap*, −ve control denotes diet only. Fot *Tc* +ve control denotes dsRNA alone; −ve controls are flour disc alone and flour disc with larvae.

The stability of dsRNA in aphid artificial diet and flour discs fed to beetle larvae was assessed to establish how often diets needed to be replaced to ensure insects were exposed to intact dsRNAs in feeding assays. Five-day old aphids were fed on diet containing 250 ng/µL *VTE* dsRNA (final volume 50 µL) and chloroform-extracted diet samples, taken at different time points, were subsequently separated on agarose gels. As shown in Figure 4A dsRNA remains intact in aphid diet for at least 24 h, whereas a reduced level of intact dsRNA is present after 48 h, and after 72 h of feeding intact dsRNA is barely detectable. Comparable analysis of dsRNA stability in flour discs (Figure 4B) shows that intact dsRNA (prominent 380 bp fragment) can be detected in the *T. castaneum* diet for up to 14 days after exposure to feeding larvae, although signals from 14-day samples were weaker than earlier time points (i.e., from 4 to 96 h). A band of lower mobility was observed in samples extracted from wheat flour, which may be attributable to the formation of complexes between dsRNA and wheat proteins. 

#### 2.3.2. Oral Delivery of dsRNAs: Phenotype and Gene Suppression 

**Figure 5:**
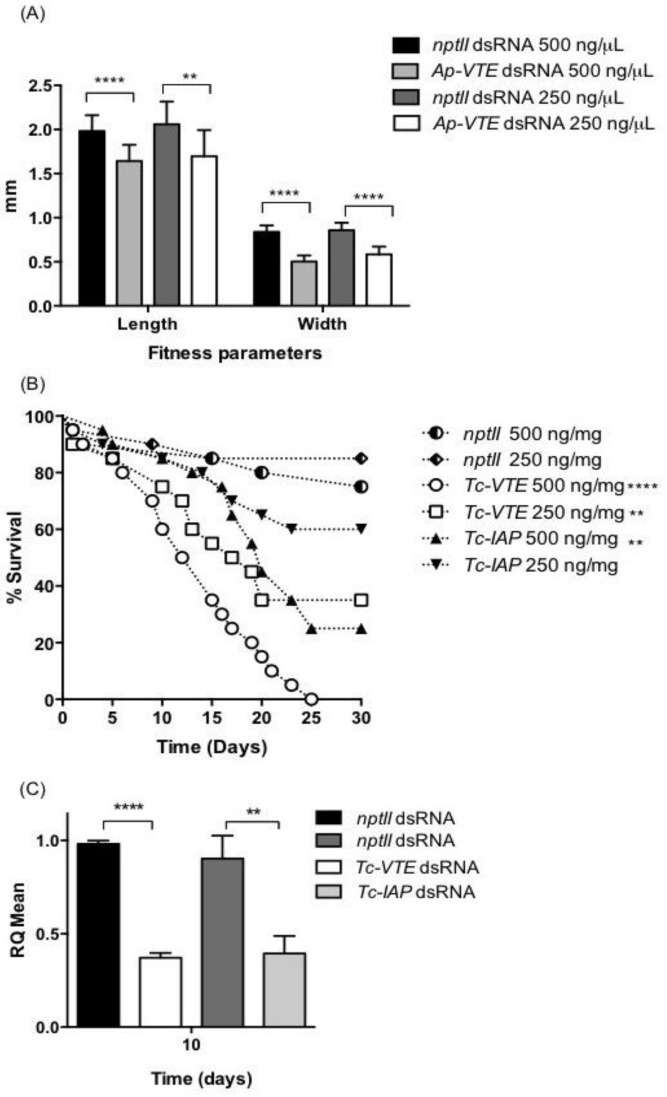
Oral delivery of dsRNAs. (**A**) Length and width of *A. pisum* 10 days after feeding on dsRNA containing diets. Asterisks depict significant differences **** *p <* 0.0001 and ** *p <* 0.01 relative to control treatments; (**B**) Survival of *T. castaneum* after feeding 1st–2nd instar larvae on flour discs containing *Tc-VTE* and *Tc-th* dsRNAs (*n =* 20 per treatment) for 30 days. Asterisks depict significant differences **** *p <* 0.0001 and ** *p <* 0.002 relative to control treatments; (**C**) Relative expression of *Tc-VTE* and *Tc-th* mRNAs in *T. castaneum* larvae after 10 days after feeding on flour discs containing dsRNAs (500 ng/mg diet). Error bars indicate the ±SD of the mean from three independent biological replicates (*n =* 5 insects per treatment), each with three technical replicates. RQ set to 1.0 for *nptII* control 10-day biological replicate 1. Asterisks depict significant differences **** *p <* 0.0001 and ** *p* = 0.0047 relative to control treatments.

On the basis of injection assays, *VTE* dsRNA was selected for oral delivery to aphids. Neonate aphids were fed on diet containing 250 ng/µL *Ap-VTE* dsRNA for 12 days, with fresh diet provided every 48 h. Survival was 100% for aphids fed on target and control dsRNAs, although aphids feeding on *VTE* dsRNA containing diets were visibly smaller than the control group. The ability of aphids to grow on dsRNA containing diets was assessed by measuring the length and width of individual aphids (*n =* 15 per treatment). As shown in Figure 5A, aphids fed *Ap-VTE* dsRNA at 500 and 250 ng/µL diet showed significant reductions in both length and width as compared to controls aphids fed with *nptII* dsRNA (*p <* 0.0001; student *t*-tests). Analysis by qPCR showed no significant down-regulation of target mRNA levels in aphids fed on diets containing *Ap-VTE* dsRNA at 500 and 250 ng/µL, as compared to controls. 

Oral delivery of *T. castaneum VTE* and *IAP* dsRNAs was carried out by feeding early stage individual larvae (≤7 days after emergence) on flour discs containing dsRNAs at 250 and 500 ng/mg diet with freshly prepared discs provided after 14 days. Survival of control larvae fed *nptII* dsRNA containing discs over the assay period was 90%. Both target dsRNA treatments caused significant mortality as compared to the control treatments (Figure 5B; *p <* 0.002, ANOVA Log-Rank Mantel-Cox tests). *Tc*-*VTE* dsRNA was the most effective treatment, causing 100% and 55% mortality as compared to 70% and 40% for *Tc*-*IAP* dsRNA at respective dietary concentrations of 500 and 250 ng/mg diet. To confirm that reduced *T. castaneum* survival was attributable to gene suppression, samples of larvae that had been fed on discs containing 500 ng/mg *Tc-VTE* or *Tc-IAP* dsRNA for 10 days were analyzed by qPCR. Transcript levels of both target genes were significantly reduced, by approx. 50% in larvae fed on dsRNA diets as compared to controls (Figure 5C; *p <* 0.0047, students *t*-tests). 

### 2.4. In Vitro Stability of dsRNA 

#### 2.4.1. Variable Persistence of dsRNAs in Insect Haemolymph

**Figure 6:**
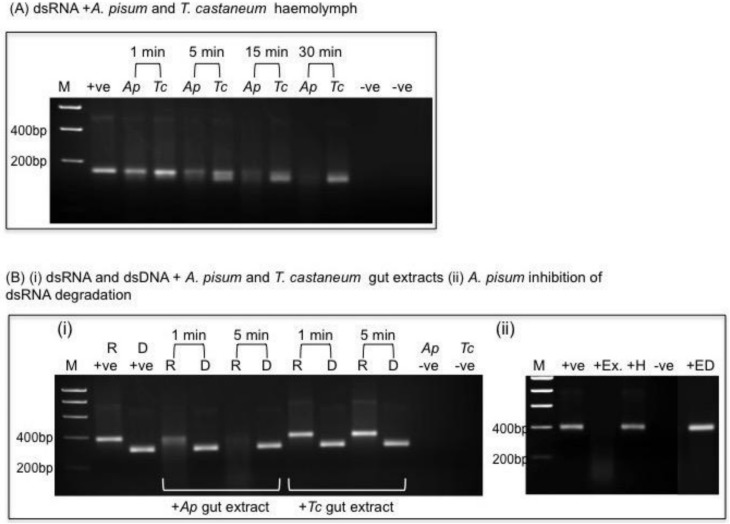

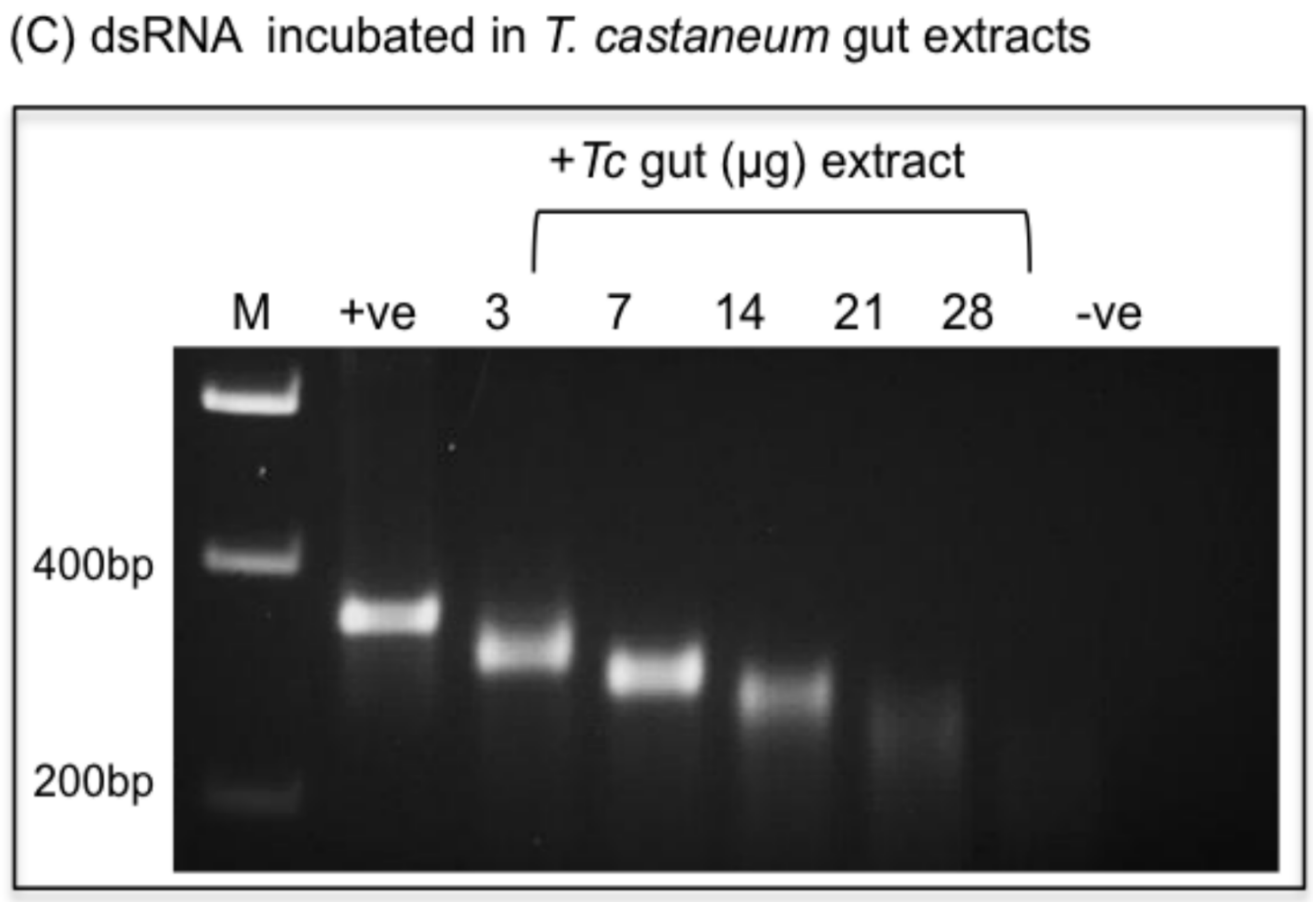
In vitro stability of dsRNAs. (**A**) dsRNA (200 ng) incubated in the presence of *A. pisum* (*Ap*) or *T. casteneum* (*Tc*) cell free haemolymph (25 µg protein); −ve controls are *Ap* or *Tc* haemolymph alone. (**B**) (**i**) 200 ng dsRNA (R) or dsDNA (D) incubated with 3 µg *Ap* or *Tc* gut extract for 1 and 5 min. +ve denotes dsRNA and dsDNA control (i.e., no added protein), −ve control is gut extract alone. (**B**) (**ii**) Inhibition of dsRNA degradation in *Ap* gut extract. Samples were incubated for 5 min at 25 °C; +ve control is dsRNA in MOPS buffer; +Ex is dsRNA incubated with 3 µg *Ap* gut protein; +H is dsRNA incubated with heat treated (65 °C for 10 min) *Ap* gut extract (3 µg protein); +ED is dsRNA incubated with gut extract in MOPS buffer with 20 mM EDTA. (**C**) dsRNA (200 ng) incubated for 30 min with increasing amounts of *T. castaneum* gut protein (as denoted).

Double stranded RNAs exhibited differences in their ability to persist as intact molecules when incubated in cell free haemolymph extracted from aphids or beetle larvae. Figure 6A shows that dsRNA is rapidly degraded in *A. pisum* haemolymph with only a faint band corresponding to intact dsRNA visible on gel after an incubation period of just 5 min. When the same amount of dsRNA is incubated in *T. castaneum* larval haemolymph (containing an equivalent amount of total protein to *A. pisum*) two dsRNA fragments are present after an incubation period of 5 min. This result is indicative of exonuclease activity, as the smaller fragment is still present after an incubation period of 30 min, whereas only a faint smear exists in the comparable aphid treatment. 

#### 2.4.2. Variable Persistence of dsRNAs in Insect Gut Extracts

Initial in vitro assays to assess the stability of 200 ng dsRNA in the presence of 3 µg of aphid or 3 µg beetle larval gut protein extracts (equivalent to 50% and 20%, respectively of total protein present per insect gut) showed that dsRNA was almost completely degraded in aphid gut extracts after an incubation period of just 1 min whereas dsRNA remained intact for up to 30 min in the presence of *T. castaneum* gut proteins. Subsequently the specificity of nuclease activity was investigated by incubating dsRNA and dsDNA in the presence of aphid and beetle gut extracts. As shown in Figure 6B(i) degradation of dsRNA in the presence of *A. pisum* gut extract (3 µg protein) is apparent after just 1 min with complete degradation observed after 5 min. By contrast, dsRNA remains intact in the presence of *T. castaneum* gut extract (3 µg protein) after an incubation period of 5 min. In both aphid and beetle samples dsDNA remained intact when incubated with gut extracts for 5 min at 25 °C. Degradation of dsDNA in the presence of aphid or beetle gut extracts was observed in subsequent experiments where higher amounts of protein (25 µg) were used. The addition of the chelating agent Ethylenediaminetetraacetic acid (EDTA) or pre-heating gut extract both inhibited dsRNA degradation in *A. pisum* gut extracts (Figure 6(Bii)) providing evidence that heat-labile metal-dependent ribonucleases are responsible for dsRNA degradation.

The stability of dsRNA to degradation in *T. castaneum* guts was further investigated by incubating dsRNA (200 ng) for 30 min in the presence of increasing amounts of gut extract which, as shown in Figure 6C, was found to result in the appearance of dsRNA fragments of decreasing size. These results indicated that exonuclease activity was prevalent in *T. castaneum* gut extracts as opposed to endonuclease activity in *A. pisum*. 

## 3. Discussion

A systematic study has been conducted to compare RNAi effects in *T. castaneum* and *A. pisum* following the delivery of dsRNAs of identical length targeting two genes known to be critical for insect development. Membrane-bound V-type ATPase proton pumps that play a vital role in nutrient uptake and ion balance in the insect gut are ideal targets for RNAi [54,55] and a number of previous studies have shown significant RNAi effects by targeting expression of genes encoding the E or A enzyme sub-units [3,33,56,57]. Similarly, the control of apoptosis is vital for development and RNAi studies targeting the expression of *inhibitor of apoptosis* (*IAP*) genes have previously been reported in dipteran and hemipteran species [58,59,60]. 

Endogenous *V-type ATPase E* (*VTE*) transcript levels in *T. castaneum* were found to be highest during the pupal stage, when a large amount of energy is required to support metamorphosis, and this is consistent with a report that V-type ATPase subunit A mRNA levels peak in the pupal stage of the small hive beetle (*Aethina tumida*) [41]. In contrast to *A. tumida*, high *VTE* transcript levels are also seen in adult *T. castaneum*. Fu et al. [57] also found high *VTE* mRNA levels in Colorado potato beetle adults (*Leptinotarsa decemlineata*) whereas lowest expression occurred in the pupal stage. In *A. pisum*, *VTE* mRNA levels were readily detected throughout the life cycle. For *IAP*, transcript levels in *T. castaneum* were highest in eggs and pupae, with relatively low levels detected in larvae and adults. This is comparable to *IAP* expression profiles previously reported for two dipteran species (*Musca domestica* and *Delia radicum*) and highlights the importance of the role of IAP during the metamorphic pupal stage [60]. As for *VTE*, *IAP* transcripts were readily detectable throughout the life-cycle of *A. pisum*. This contrasts variable expression profiles for *IAP* during the life-cycle of the hempiteran tarnished plant bug (*Lygus lineolaris*) where relatively low levels were detected in nymphs and highest expression in adults [61]. Thus, the expression of developmentally critical genes during the development of different insect species even within the same order can be highly variable.

Pre-pupal *T. castaneum* were injected with dsRNAs on the basis that this was the developmental stage at which relatively high levels of target mRNAs were present. Five-day-old aphids were injected with dsRNAs as target transcripts were similarly abundant throughout the life-cycle and this is the earliest stage at which injection is feasible. Injections of 50 and 100 ng of *VTE* or *IAP* dsRNAs resulted in similarly significant dose dependent reductions in the survival of beetle larvae. Levels of >85% gene down-regulation, as compared to controls were also similar for the two gene targets at both 48 h and 10 days post injection indicative of a stable and systemic RNAi response in *T. castaneum.* Systemic RNAi induced by injection of dsRNA in *T. castaneum* larvae has been validated in previous studies that show significant target gene suppression and RNAi-specific phenotypes [6,7,9,62]. Furthermore, RNAi effects have been detected throughout larvae rather than being localized to the site of injection [5], and effects have also been detected in offspring embryos [4].

Dose dependent reductions in survival were observed for aphids injected with *VTE*, but not *IAP* dsRNAs; although aphid mortality was only significantly different to the control treatment at the highest injection dose of 30 ng (comparable on a per mg insect to 100 ng injected into *T. castaneum*). *V-type ATPase E* transcript levels were lower than controls (approx. 40%) 24 h post injection, but recovered to control levels after 72 h demonstrating that RNAi effects in *A. pisum* were weak and transient. Unlike *T. castaneum*, injections of *IAP* encoding dsRNAs did not induce significant aphid mortality nor reduced transcript levels. Possamai et al. [63] reported a 30–40% reduction in calreticulin and gut specific *cathepsin-L* transcript levels following injection of *A. pisum* with approx. 270 ng of dsRNAs. As observed for *IAP* in this study, the injection of 80 ng dsRNAs targeting a molting hormone receptor gene failed to induce any measurable effect towards *A. pisum* [43]. 

The stability of dsRNAs in beetle and aphid diets was evaluated to ensure insects were continuously exposed to dsRNAs during feeding assays. Flour beetles are not known to secrete extra-orally to facilitate digestion and thus it was not surprising to find that dsRNA was stable in dried flour discs for up to 14 days. By contrast, dsRNA remained intact in the presence of feeding *A. pisum* for only 24–48 h, and was fully degraded after 72 h of feeding. This result is comparable with Christiaens et al. [43] who reported degradation of dsRNA in diet in the presence of *A. pisum* after 84 h. Rapid degradation of dsRNA in the presence of saliva and salivary gland extracts has also been reported for *Lygus lineolaris* (tarnished plant bug) and the southern green stinkbug (*Nezara virridula*) [51,52].

Feeding early stage *T. castaneum* larvae on dsRNAs resulted in dose dependent reductions in survival and gene down regulation. As for injection assays, higher levels of mortality were obtained for *VTE* treatments as compared to *IAP* treatments although levels of gene knock down (ca. 60%) were similar for both transcripts. Differences in survival could thus be due to the higher and more consistent presence of *VTE* transcripts, reflecting the essential role that the enzyme plays throughout larval development, as compared to *IAP* transcripts, which are expressed at relatively low levels in larvae. The highly efficient RNAi response in *T. castaneum* has previously been demonstrated in studies where feeding larvae just 2.5 ng *VTE* dsRNA/mg diet for 7 days resulted in 50% mortality [33]. In this study we were interested in making a direct comparison of RNAi efficiency in a susceptible (*T. castaneum*) and recalcitrant (*A. pisum*) insect species. Our beetle results are comparable to Halim et al. [34] who recorded significant mortality (19–51%) of late stage *T. castaneum* larvae fed for 6 days on flour discs containing dsRNA (50–150 ng/mg diet) targeting the expression of *voltage–gated sodium ion channel* transcripts. 

No mortality was observed after feeding *A. pisum* nymphs to maturity on diets containing up to 500 ng/µL dsRNAs, although *VTE* dsRNA (at doses of 500 and 250 ng/µL diet) did cause a significant reduction in growth and fecundity. Whilst a phenotype was observed, no evidence for gene down regulation was obtained and this may be attributable to the level of down regulation being too little or transient to be detected, and/or fitness costs associated with dsRNA degradation. A few studies have reported successful RNAi in pea aphids after feeding dsRNAs although difficulties have also been reported more generally for Hemipteran species including *A. pisum* [42]. Here we conclude that RNAi effects in pea aphids are, at least in part, dependent upon the gene target. 

Injection and feeding studies showing persistent and systemic RNAi effects in *T. castaneum* versus weak and transient effects in *A. pisum* correlate with differences in the stability of dsRNAs in the presence of cell free haemolymph and gut extracts. Double-stranded RNA remained largely intact when incubated in *T. castaneum* haemolymph for up 30 min, although partial digestion was indicated by the reduced mass of the dsRNA detected by fluorescence on agarose gels. By contrast, signs of dsRNA degradation in the presence of aphid haemolymph were apparent after just 5 min of incubation and full degradation after 30 min. In gut assays dsRNA was degraded within 1–5 min of incubation with *A. pisum* gut extracts whereas it remained intact for up to 30 min in comparable *T. castaneum* samples. This finding is in agreement with Singh et al. [53], who reported that the concentration of body fluid (including lumen and haemolymph) required to degrade 50% of dsRNA in *T. castaneum* was 4.68 mg/mL and only 0.07 mg/mL in *A. pisum*. Furthermore, Singh et al. [53] reported that processed siRNA from dsRNA could be detected in total RNA from dsRNA injected/fed *T. castaneum*, but not from *A. pisum*. Of note here is that dsRNA shows a distinct gradual reduction in size (bp) in the presence of increasing amounts of *T. castaneum* gut extract which is indicative of exonuclease rather than endonuclease activity. By contrast, in *A. pisum* the rapid and complete disappearance of dsRNA in haemolymph and gut extracts could due to degradation by endonucleases and/or exonucleases. The RNase responsible for dsRNA degradation in *A. pisum* gut extracts was shown to be heat labile, metal dependent and inactivated by the presence of EDTA.

The persistence of dsRNA in the insect extracellular environment is crucial for cell uptake of dsRNA and the subsequent induction of RNAi. Nucleases that may be responsible for reducing the efficiency of RNAi in insects due to their ability to rapidly degrade dsRNAs have been identified. Arimatsu et al. [64] identified a non-specific DNA/RNA nuclease (BmdsRNase) in silkmoth (*Bombyx mori*) larvae that was secreted from midgut epithelial cells into the gut lumen. Homologous dsRNase sequences have subsequently been identified in *A. pisum* (ApDsNucl1 and ApDsNucl2) by Christiaens and Smagghe [43] and more recently in *T. castaneum* (Tc_dsRNase1 and Tc_dsRNase 2) [47]. An exonuclease Rrp44-like protein (XP_001601829) with potential responsibility for dsRNA degradation has been identified in the salivary gland of the starnished bug (*N. vitripennis*) [52]. The identified protein contains a PIN_Rrp44 domain, which is known for its endonuclease activity and 3′-5′ exoribonuclease activity in the yeast *Sacchromyces cerevisiae* [65], as well as an exoribonuclease R domain, which is broadly distributed throughout the bacteria [66]. According to our results, a highly processive hydrolytic 3′–5′ exonuclease may be responsible for the observed degradation of dsRNA in *T. castaneum* gut extracts and we have identified a homologous Rrp44-like (LOC655788) sequence in *T. castaneum*, which shares 49% homology with the PIN_Rrp44 domain and 69% with the Exoribonuclease R domain of the nuclease Rrp44-like protein in tarnished bug. 

Rapid degradation of dsRNAs due to nuclease activity in the saliva, haemolymph and guts of *A. pisum*, and more generally hemipteran species, could be an adaptive evolutionary response to a heavy viral loads leading to constitutive expression of active nucleases against viral RNAs or plant defence nucleic acids [43,45,52]. Here, we provide further evidence that, not only are the levels of nuclease activity different between *T. castaneum* and *A. pisum*, but also the nature of ribonucleases is different. We suggest that the slow, progressive degradation of dsRNA in *T. castaneum* is due to exonuclease activity, and that relatively limited nuclease activity in the extracellular environment essentially allows sufficient time to allow cellular uptake. By contrast, rapid and complete degradation of dsRNA by extracellular exo- and endo-nucleases may well be the primary factor in limiting RNAi effects in *A. pisum*, and in hemipterans generally.

## 4. Materials and Methods 

### 4.1. Insects

*Acyrthosiphon pisum* were maintained on broad bean plants (*Vicia faba*) at 25 °C, under a lighting regime of 16 h L: 8 h D. Adults were collected and transferred from plants to chambers containing artificial diet [67] to obtain neonate (0–24 h) nymphs which were collected for feeding assays using a paint brush. *Tribolium castaneum* larvae and adults were reared continuously in whole organic flour containing 5% (*w*/*w*) brewer’s yeast at 25 °C, under a lighting regime of 16 h L:8 h D with 75% relative humidity. For feeding assays, flour was passed through a sieve (aperture size 300 µm; Glenammer Engineering) in order to separate larvae and eggs. 

### 4.2. Cloning of VTE and IAP Gene Sequences for dsRNA Production

Insects were snap frozen in liquid nitrogen and total RNA extracted using a Quick-RNA^TM^ Miniprep kit (ZYMO Research, Irvine, CA, USA), according to the manufacturer’s protocol. Total RNA was quantified by using Nano-drop spectrophotometer (Model ND-1000, Thermo Scientific, Waltham, MA, USA). Synthesis of cDNA was performed from 1 μg total RNA using a mixture of oligo-d(T) and random hexamer primers from SensiFAST^TM^, and a cDNA synthesis kit (Bioline, London, UK) as described in the manufacturer’s protocol.

Primers were designed to amplify PCR products of 277 bp for both *V-type ATPase E subunit* (*T. castaneum*: Acc. No. XM_965528; *A. pisum*: Acc. No. XM_001946489) and *inhibitor of apoptosis* (*T. castaneum*: Acc. No. XM_969968.2; *A. pisum*: Acc. No. XM_001944122.3) transcripts from 200 ng cDNA template. All cloning primers (Integrated DNA Technologies, Available online http://www.idtdna.com/CodonOpt) including restriction enzyme sites (*Xhol* and *Xbal*) (Fermentas, Waltham, MA, USA) are listed in Table 1. A kanamycin-resistance gene (*nptII*) 600 bp sequence was amplified from the plasmid PSC-A-amp/Kan vector (Agilent Technologies, Santa Clara, CA, USA) as a negative control. PCR amplification was performed using Phusion High-Fidelity DNA polymerase (Thermo Scientific) with conditions as follows: 98 °C for 30 s, followed by 15 cycles of 10 s 98 °C, 30 s at 58 °C and 30 s at 72 °C, with a final extension step of 72 °C for 10 min. Amplified PCR products were electrophoresised on 1% DNA agarose gels and extracted using QIAquick columns (Qiagen, Hilden, Germany). Eluted PCR products were ligated into pJET1.2 vector (CloneJET PCR cloning kit, Thermo Scientific Life Science Research) following the manufacturer’s protocol. Sequences of recombinant plasmids were confirmed by DNA sequencing.

### 4.3. Production of dsRNA: In Vitro Transcription 

Plasmids containing target templates were linearized with either *XhoI* or *XbaI* for production of sense and antisense dsRNA strands and ethanol precipitated. Sense and antisense RNA were synthesised in vitro using T7 RNA polymerase (Megascript T7 transcription kit, Ambion, Waltham, MA, USA) and 1 μg of linearised DNA template in a total volume of 20 μL. Remaining DNA templates in the reactions were removed by DNase digestion. Single-strand RNAs were purified by phenol chloroform extraction, ethanol precipitated and re-suspended in nuclease free water. Equal amounts of sense and antisense single stranded (ss)RNAs were mixed and then annealed by heating at 85 °C for 5 min and then slowly cooled to room temperature.

### 4.4. Analysis of Gene Expression by Quantitative PCR

Total RNA was isolated from pooled samples of insects (5 insects per biological replicate) after injection or feeding using a Quick-RNA^TM^ Miniprep kit (ZYMO Research). DNA contamination was removed by DNase digestion and the quality of isolated RNA was validated by Nano-drop. cDNA synthesis was performed using a SensiFAST^TM^ cDNA synthesis kit (Bioline) as described above. Quantitative real-time PCR experiments were performed according to the MIQE guidelines outlined by Bustin et al. 2013, using a 96 well ABI Step one Plus real-time PCR instrument and GoTaq^®^ qPCR Master Mix (Promega, Madison, WI, USA) with comparative CT methodology. CXR was used as reference dye in each reaction. Gene expression was normalised to *GAPDH* with triplicates performed for each biological replicate sample. Primers listed in Table 1 were designed using ABI primer express software for real-time PCR. 

### 4.5. Expression of VTE and (IAP) during the Life Cycle of T. castaneum and A. pisum 

The expression profile of target genes throughout the life cycle of *A. pisum* and *T. castaneum* was assessed by quantitative PCR. Under insectary conditions, the life cycle of *A. pisum*, from neonate to maturity (onset of nymph production) takes 10–13 days, whereas it takes approx. 40 days for *T. castaneum* to develop from hatch to mature adult. Total RNA was extracted from 5-pooled insects or approx. 30 mg weight of *T. castaneum* eggs, and used to prepare cDNA. Expression of the target genes was estimated relative to an endogenous control (*GAPDH*) by quantitative PCR (qPCR).

### 4.6. Delivery of dsRNA to Insects

#### 4.6.1. Injection

Injections of dsRNAs into 5-day old *A. pisum* nymphs (approx. 0.8 mg weight) were carried out using a Nanoject II^TM^ injector (Drummond Scientific Company, Broomall, PA, USA) under a dissecting microscope (SX-45, Vision). Aphids were anaesthetised with CO_2_ for 2 min prior to injection. Doses of 7.5 to 30 ng of *A. pisum VTE* (*Ap-VTE*) or *A. pisum IAP* (*Ap-IAP*) dsRNAs were injected into the ventral abdomen and aphids were subsequently placed on artificial diet. Control aphids were injected with 30 ng *nptII* dsRNA. Survival was monitored for a minimum of 7 days post injection. Samples were collected 24 h, 72 h and 6 days after injection for analysis of gene expression by qPCR. 

Double stranded RNAs were injected as described by Tomoyasu and Denell (2004) into the dorsal side of *T. castaneum* larvae (approx. 3.3 mg weight). Fifty nanograms of *Tc-VTE* or *Tc-IAP* dsRNAs were injected into pre-pupa stage larvae. Larvae were collected 48 h and 10 days post injection for analysis of gene expression by qPCR. 

#### 4.6.2. Feeding

*Ap-VTE* and *nptII* dsRNAs were fed to neonate aphid nymphs by mixing dsRNAs in artificial diet to a final concentration of 250 ng/µL and 500 ng/µL. Fresh diet was provided every 2 days during bioassays and nymphal survival and development was monitored daily for 12 days. In addition the size (length and width) of aphids were recorded and analysed by using Image J [68] after 10 days. The stability of dsRNA in aphid diet was assessed by analysing diet upon which aphids had fed. To this end fifteen 5-day old aphids were placed on diet containing 500 ng/µL *Ap-VTE* dsRNA, and 20 µL of diet was collected after 4 h, 24 h, 48 h and 72 h of feeding. A control sample not exposed to aphids was also included. Diet samples were extracted with phenol:chloroform:isoamyl alcohol prior to separation on 2% (*w*/*v*) agarose gels.

For *T. castaneum* feeding assays, *Tc-VTE* dsRNA and *Tc-IAP* dsRNAs were delivered using flour discs prepared as described by Xie et al. [69]. Double stranded RNAs (*nptII*, *Tc-VTE* and *Tc-IAP*) in 200 µL of nuclease-free water were mixed with 50 mg of sieved wholewheat organic flour containing 5% (*v*/*v*) yeast. Ten microlitres of the mixture was added to individual wells of a 96-well flat-bottomed microtitre plate and allowed to dry for 8 h in a Laminar flow hood. The final concentration of dsRNAs was 250 and 500 ng per mg of flour disc. A single first instar (<1-week-old) *T. castaneum* larva was added to each well and survival was assessed for 30 days. Larvae were collected after 5 days of feeding for qPCR analysis. The stability of dsRNA in *T. castaneum* diet was evaluated by analysing diet upon which larvae had fed. One-week-old individual larvae were fed on flour discs containing 500 ng/mg *Tc-IAP* dsRNA for 4 h, 24 h, 48 h, 72 h, 96 h, 120 h and 14 days. Diet samples were subsequently re-dissolved in 30 µL nuclease-free water, phenol:chloroform:isoamyl alcohol extracted and separated on 2% (*w*/*v*) agarose gels.

### 4.7. In Vitro Stability of dsRNA in Insect Tissues

#### 4.7.1. Tissue Extract Preparation

Haemolymph samples were collected in ice cold 1× phosphate buffered saline (PBS, pH 7.4) in microfuge tubes containing 1 mg phenylthiourea (PTU) to prevent melanisation [70]. To collect haemolymph from *A. pisum*, the legs of the aphid were removed and the body squeezed gently using forceps to allow collection of exuded haemolymph. For *T. castaneum* larvae, a fine steel needle was used to pierce the skin, and exuded haemolymph was collected. Haemocytes were removed by centrifugation at 17,500× *g* for 30 min at 4 °C. Insect gut extracts (including contents) were prepared by extraction in 1× PBS. Forceps were used to separate the head (larvae or aphid) and attached gut from the insect body. Gut samples were then homogenized in a micro-pestle, followed by centrifugation as described above. The concentrations of total protein in haemolymph samples and gut extracts were estimated by BCA assay using BSA as a standard protein. 

#### 4.7.2. In Vitro dsRNA and dsDNA Gut Stability Assays

The stability of dsRNA in insect gut extracts was initially investigated by incubating dsRNAs with different amounts of total gut protein. For *A. pisum*, 200 ng dsRNA was incubated for 30 min at 25 °C in the presence of 1–10 µg of total gut protein in a 20 µL reaction. For *T. castaneum*, 200 ng dsRNA was incubated for 30 min at 25 °C with 3–28 µg of total gut protein. Double stranded RNA in 1× PBS was used as positive controls and gut samples alone as negative controls. After incubation, the integrity of the dsRNA was analysed on 2% (*w*/*v*) agarose gels.

Subsequent assays assessed dsRNA stability with time (1–30 min at 25 °C) in the presence of comparable amounts (3 µg) of total *A. pisum* or *T. casteneum* gut protein. In addition, the stability of dsDNA and dsRNA to degradation was compared by incubating 3 µg of gut protein with 200 ng nucleotides for 1 min and 5 min at 25 °C. Double stranded RNA or DNA in 1× PBS were used as positive controls and gut samples alone as negative controls. Stability to heat treatment was assessed by incubating 3 µg *A. pisum* gut extract in 20 µL reactions that had previously been heated to 65 °C for 10 min. The ability of EDTA to inhibit degradation was evaluated by the addition of 20 mM EDTA to a 20 µL sample containing 200 ng dsRNA and 3 µg *A. pisum* gut extract (40 mM MOPS buffer; pH 7.5). After incubation, the integrity of the dsRNA or dsDNA was analysed on 2% (*w*/*v*) agarose gels.

#### 4.7.3. In Vitro dsRNA Haemolymph Stability Assays

The stability and persistence of dsRNA in aphid and larval haemolymph samples was investigated in a similar manner to in vitro gut assays. Double stranded RNAs (200 ng) were mixed with *A. pisum* or *T. castaneum* haemolymph samples containing 25 µg protein in 20 µL reactions incubated at 25 °C for 1–30 min. Double stranded RNA incubated with 1× PBS containing PTU were used as positive controls and haemolymph samples only as negative controls. After incubation, the integrity of the dsRNA was analysed on 2% (*w*/*v*) agarose gels.

### 4.8. Statistical Analysis

The qPCR results are reported as mean ± SD of three independent biological replicates and differences of gene expression between treatments were compared by student’s *t*-test. Survival curves were compared using Log-rank Mantel-Cox tests. All statistical analyses were performed using GraphPad Prism version 6.0 with *p* < 0.05 considered significant.

## Figures and Tables

**Table 1:** Sequence of forward (F)/reverse (R) primers used for cDNA sub-cloning, dsRNA synthesis and qPCR analysis. *Tc* denotes *T. castaneum* and *Ap* denotes *A. pisum*.

Gene (Accession No.)	Insect Species	Sequence 5′-3′
*V-type ATPase E subunit * (XM_965528)	*Tc*	cDNA sub-cloning and in vitro transcription: F: TATCTCGAGACCAGGCGAGATATTCACAGC R: TATCTCGAGAAACGAGCCTCCAAGGTGTTG
		qPCR analyses: F: CCAAGCATTTTTAATGCA CCAC R: AACCACCACGACCTTGAATAG
*Inhibitor of Apoptosis * (XM_969968.2)	*Tc*	cDNA sub-cloning and in vitro transcription: F: ATATCTAGAAGTTCGGCTGTAACTCCCG R: ATACTAGACATCCGGAACGTCTCACTCT
		qPCR analyses: F: AAGCGAAAAGTTGAGGCAAGC R: AACCATTGCTTTCTTACTCGAAGG
GAPDH (XP_974181.1)	*Tc*	qPCR analyses: F: CCGGGATGGCGTTCAG R: CCAAACGCACCGTCAAATC
*V-type ATPase E subunit * (XM_001946489)	*Ap*	cDNA sub-cloning and in vitro transcription: F: TATCTCGAGGGGCCGCCTGGT R: ATATCTAGACACGAACACGTAATGTGA
		qPCR analyses: F: CCGAGTATAAGGCAGCATCCA R: CTTATGTGCCAACAACTCAATACCA
*Inhibitor of Apoptosis * (XM_001944122.3)	*Ap*	cDNA sub-cloning and in vitro transcription: F: TATCTCGAGGGTCTGAAGGACTGGGAAGAA R: GCTTCCGGCGTAGGTGTTCTAGAATA
		qPCR analyses: F: GATTATTGGCAACAAGGTGATGATC R: AACCAGCAGAAGAATCGTTAAAAAA
GAPDH (NM_001293474.1)	*Ap*	qPCR analyses: F: CAATGGAAACAAGATCAAGGTGTT R: ACCAGCAGATCCCCATTGG
*Kanamycin resistance* (JN638547)	-	F: AGGCTATTCGGCTATGAC R: CGATAGAAGGCGATGCG
